# Complete genome sequence of Mulvp2, an *Enterobacter* bacteriophage

**DOI:** 10.1128/mra.01004-25

**Published:** 2025-12-16

**Authors:** Zhaoxia Dong, Zhixian Zhu, Feng Zhang, Cheng Zhang, Cui Yu

**Affiliations:** 1 Industrial Crops Institute of Hubei Academy of Agricultural Sciences, Wuhan, Hubei, China; Queens College Department of Biology, Queens, New York, USA

**Keywords:** *Enterobacter mori*, phage therapy, phage genome

## Abstract

Herein, we present Mulvp2, a bacteriophage isolated from mulberry field soil. Mulvp2 infects *Enterobacter mori* and has a siphoviral morphology. Mulvp2 has a 58,539 bp dsDNA genome with 72 annotated genes and a GC content of 50.23%.

## ANNOUNCEMENT

Bacteriophages are viruses that specifically infect bacteria and are of great value for the prevention and treatment of bacterial infections in fields such as healthcare, food, agriculture, and the environment (1–3). The isolation and characterization of bacteriophages are fundamental for the construction and application of bacteriophage resource libraries. Herein, the bacteriophage Mulvp2 was isolated using the bacterial host *Enterobacter mori*, which causes bacterial wilt and soft-rot diseases in many crops (4–6).

**Fig 1:**
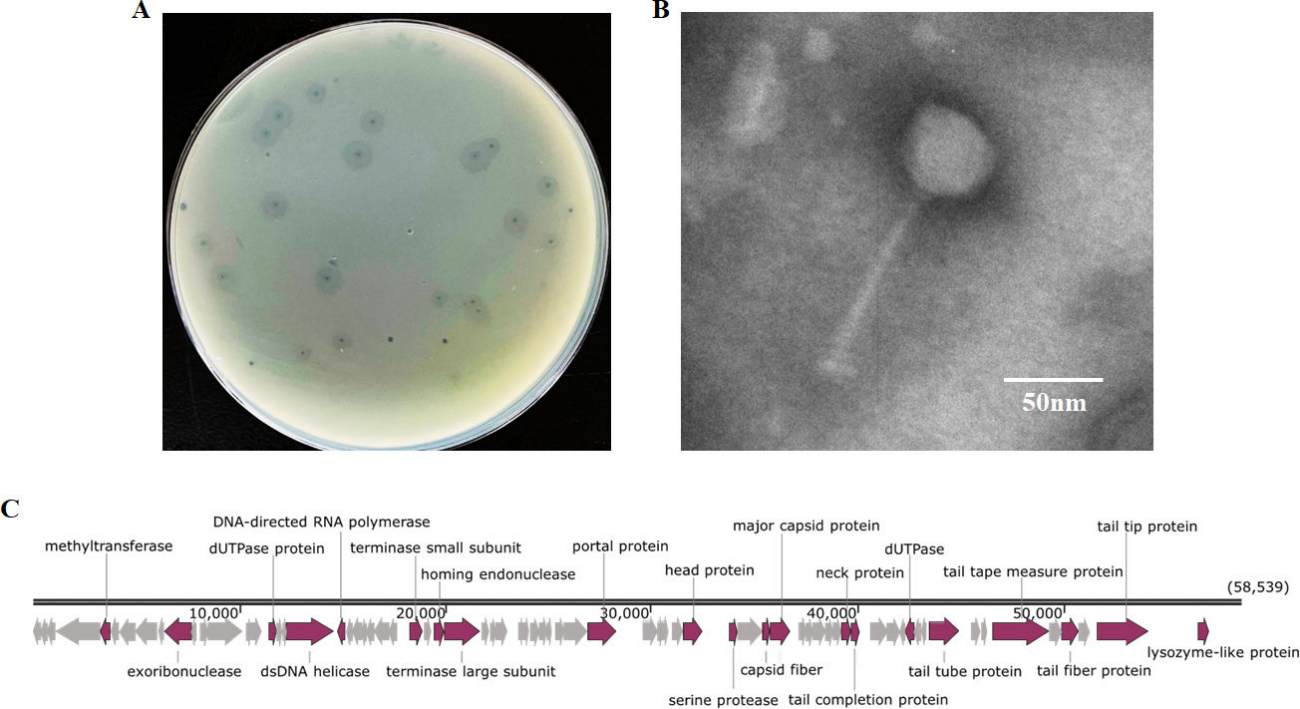
Characterization of the phage Mulvp2. (**A**) Plaques of Mulvp2 formed on the double-layer agar plate. (**B**) Morphology of phage particles. Phage particles were observed using transmission electron microscopy via negative staining with 2% phosphotungstic acid (pH 6.8), and the magnification was ×40,000. Scale bar: 50 nm. Phage particle measurements are shown in Table 1. (**C**) Structure map of the Mulvp2 genome. Arrows indicate the direction of transcription for predicted ORFs.

Mulvp2 was isolated from the soil of mulberry fields in the city of Wuhan, Hubei Province, China (114.316337°N, 30.478924°W), in 2022, using a previously described enrichment method with minor modifications (7). Briefly, 5 g of the collected soil was added to the phage buffer (50 mM Tris-HCl [pH 7.5], 100 mM NaCl, 8.1 mM MgSO₄, and 0.01% gelatin) and incubated at 28°C with shaking at 220 rpm for 12 h. This mixture was centrifuged (13,000 × *g*, 10 min), and the supernatant was passed through a 0.22 μm sterile filter; the sterile filtrate was mixed with *Enterobacter mori* GQN5-3 culture and semisolid BG medium (1% peptone, 0.1% yeast extract, 0.1% casamino acids, 0.5% glucose, and 0.8% agar). The mixture was then poured into plates containing solid BG medium at the bottom and incubated at 28°C for 12–24 h until clear phage plaques formed. Purified phage was obtained after three rounds of purification. The plaque assay indicated that Mulvp2 produced small, clear plaques surrounded by enlarged halos (Fig. 1A), which is characteristic of plaques produced by phages capable of degrading bacterial exopolysaccharides (8, 9). Mulvp2 morphology was observed using transmission electron microscopy (FEI Tecnai G2 20 TWIN, 200 kV), which revealed a siphoviral morphology with an icosahedral head and a noncontractile tail (Fig. 1B; Table 1).

**TABLE 1:** Genomic contents of the bacteriophage Mulvp2

Parameter	Result for Mulvp2
	
Sample characteristics	
Collection location coordinate	114.316337°N, 30.478924°W
Culture temperature	28°C
Phage capsid diameter (nm)	49.07 ± 1.35 nm (*n* = 10)
Phage tail length (nm)	104.17 ± 3.2 nm (*n* = 10)
Sequencing	
Sequencing instrument	Illumina Novaseq
Library prep kit	TruSeq DNA Sample Prep Kit
Number of reads	12,614,312
Length of reads	150 bp paired-end reads
Shotgun coverage (×)	32,321
Phage genome characteristics	
Genome length (bp)	58,539
Genome character	Linear
Genome termini type	Circularly permuted
GC content (%)	50.23
Number of identified genes	72
Number of genes with possible function	21
Number of ncRNA	0
Most similar phage	TayeBlu (93.3% identity with a query cover of 68%)

Genomic DNA was prepared from freshly harvested phage suspensions using the zinc chloride precipitation method (10). Sequencing of phage genomic DNA was performed on the Illumina NovSeq sequencing platform using the TruSeq DNA Sample Prep Kit, generating 12.6 million paired-end 150 bp reads with 32,321-fold coverage. Fastp v.0.20.0 was used for quality control of the sequencing data (11). Abyss v.1.5.4 (12) and SPAdes v.3.12.0 (13) were used for phage genome assembly, and Pilon v.1.18 (14) was used for single-base correction to obtain the final bacteriophage genome sequence. PhageTerm was used to identify the phage genome termini (15). Protein-coding genes in the Mulvp2 genome were predicted using GeneMarkS v.4.32 (16), while sequence alignment of the encoded proteins against the NCBI nonredundant protein database was conducted using DIAMOND v.0.8.36 (17). Default parameters were used for all software unless otherwise specified.

The genomic characteristics of Mulvp2 are shown in Table 1. Mulvp2 has a 58,539 bp dsDNA genome with a GC content of 50.23%. The Mulvp2 genome was predicted to encode 72 genes, 21 of which had possible functions. Rfam database (18) search revealed no noncoding RNAs or tRNAs in the Mulvp2 genome. Genomic comparisons revealed that the *Azotobacter* phage TayeBlu is the phage most similar to Mulvp2 (Table 1).

## Data Availability

Sequence data for phage Mulvp2 are available in GenBank with accession no. OR508996 and Sequence Read Archive no. SRX30365921.
